# Novel DNA Aptameric Sensors to Detect the Toxic Insecticide Fenitrothion

**DOI:** 10.3390/ijms221910846

**Published:** 2021-10-07

**Authors:** Kien Hong Trinh, Ulhas Sopanrao Kadam, Jinnan Song, Yuhan Cho, Chang Ho Kang, Kyun Oh Lee, Chae Oh Lim, Woo Sik Chung, Jong Chan Hong

**Affiliations:** 1 Division of Life Science and Applied Life Science, Plant Molecular Biology and Biotechnology Research Center, Gyeongsang National University, Jinju 52828, Gyeongnam, Korea; thkien@vnua.edu.vn (K.H.T.); ukadam@gnu.ac.kr (U.S.K.); Jinnansong93@gmail.com (J.S.); yoohan2138@gmail.com (Y.C.); jacobgnu69@gnu.ac.kr (C.H.K.); leeko@gnu.ac.kr (K.O.L.); colim@gnu.ac.kr (C.O.L.); chungws@gnu.ac.kr (W.S.C.); 2 Faculty of Biotechnology, Vietnam National University of Agriculture, Hanoi City 12400, Vietnam; 3 Division of Plant Sciences, University of Missouri, Columbia, MO 65211, USA

**Keywords:** fenitrothion, aptamer, SELEX, thioflavin T, fluorescence, insecticide

## Abstract

Fenitrothion is an insecticide belonging to the organophosphate family of pesticides that is widely used around the world in agriculture and living environments. Today, it is one of the most hazardous chemicals that causes severe environmental pollution. However, detection of fenitrothion residues in the environment is considered a significant challenge due to the small molecule nature of the insecticide and lack of molecular recognition elements that can detect it with high specificity. We performed in vitro selection experiments using the SELEX process to isolate the DNA aptamers that can bind to fenitrothion. We found that newly discovered DNA aptamers have a strong ability to distinguish fenitrothion from other organophosphate insecticides (non-specific targets). Furthermore, we identified a fenitrothion-specific aptamer; FenA2, that can interact with Thioflavin T (ThT) to produce a label-free detection mode with a *K_d_* of 33.57 nM (9.30 ppb) and LOD of 14 nM (3.88 ppb). Additionally, the FenA2 aptamer exhibited very low cross-reactivity with non-specific targets. This is the first report showing an aptamer sensor with a G4-quadruplex-like structure to detect fenitrothion. Moreover, these aptamers have the potential to be further developed into analytical tools for real-time detection of fenitrothion from a wide range of samples.

## 1. Introduction

In the modern world, insecticides are widely used to protect crop plants and control insects in rural and urban societies. Insecticides play a vital role in securing food production and improving quality of life by controlling several insect-transmitted diseases in plants, animals, and humans [1]. At present, thousands of pesticides or insecticides are commercially available; many of them carry fenitrothion as an active ingredient [1,2]. However, excessive use of fenitrothion causes environmental pollution and several hazards to wildlife and natural ecosystems, including human health.

Fenitrothion is a broad-spectrum insecticide [1,3], which belongs to the organophosphate group of small molecule chemical insecticides. Organophosphate is one of the most diverse families of pesticides; it has a yellow–brown color, garlic odor and is slightly soluble in water at room temperature. Fenitrothion was first introduced in 1959 [2,3]; it is cheaply available [4] and is mainly used to control insect pests like flies, mosquitoes, and cockroaches in agriculture, public health programs, and indoor use [5]. The mode of action of this class of insecticides includes inhibition of acetylcholinesterase enzyme, which prevents the breaking down of the acetylcholine molecules at the synapses of insect nerve cells, resulting in unstoppable signal transmission, so that insects continue to contract the muscles until death [5,6].

According to the United States Environmental Protection Agency (USEPA), in 2012, the global expenditure on insecticides was about 56 billion USD, and fenitrothion production was 15,000–20,000 tons per year [2,7]. Commercial products containing fenitrothion are available in formulations including solutions, powders, granules, oil-based sprays, and in combination with other pesticides or insecticides. Indiscriminate use of these products for insect-pest control has caused several undesirable effects on the environment. Some studies have reported the harmful effects of fenitrothion on honey bees [8], krill [9], and rats [10]. A study showed that a mixture of pesticides containing fenitrothion at very low concentrations killed 99% of leopard frog tadpoles [11]. Moreover, recent findings have shown the residues of fenitrothion were found in green beans [12], chicken embryos, and foods [2,13]. Such accumulation of toxic compounds in natural products like foods and eggs is of great concern to human and environmental safety.

The most commonly used methods to detect small molecules of toxic chemicals today are chromatographic techniques such as GC, HPLC, LCMS, etc. [14]. However, these techniques are often complicated, time-consuming, and costly. Detection of fenitrothion contamination and environmental pollution is a critical challenge for us. Currently, several methods have been developed to detect fenitrothion, but these methods face several issues due to the lack of particular molecular recognition elements (MREs). Therefore, the main objective of our study is to discover novel aptamers that can bind to fenitrothion with high specificity and sensitivity.

Aptamers are single-stranded DNA (ssDNA) or RNA molecules that can bind to a particular target [15,16]. Aptamers have many advantages over antibodies, such as durability, ease of synthesis, simple design, long-term preservation, very high reproducibility, low cost, and, unlike antibody-based assays, there is no need to use animals for their production. Aptamers can be used to detect almost any molecule, even those that have high toxicity. On the other hand, antibodies cannot be produced against toxic materials, stimulating a hypersensitive immune response. Finally, aptamers can unfold and refold (several times) to regain their original functionality even after exposure to several denaturing cycles or after treatment with extreme pH or salt or high temperatures, which is not possible using antibodies or peptides [17,18]. Since the discovery of the aptamer in 1990 [19], several aptamers have been reported for a wide range of targets from ions to macromolecules such as proteins to even single cells, bacteria, or viruses [20,21]. Several variations exist for selecting an appropriate aptamer, but most of the methods generally involve the systematic evolution of ligands by an exponential enrichment (SELEX) process [22,23,24]. 

Currently, there are several methods used to study the binding interaction between aptamer and target molecules. These methods are based on optical, electrochemical, colorimetric, or fluorescence measurements [25,26,27]. In general, the most popular method for detecting small molecules is fluorescence-based, which relies on the covalent labeling of aptamers with a fluorophore such as 6-FAM [28,29,30]. However, such covalent modification of aptameric ssDNA may affect its stability and induce conformational changes, interfering with sensitivity and binding with small molecule targets. Therefore, chemical displacement-based assays that do not need covalent modifications are highly desired. In this study, we employed a ThT displacement assay, where ThT dye has a high affinity for G4-quadruplex DNA [31]. Upon binding to G4-quadruplex DNA [32,33], the ThT molecules fluoresce, and the change in the signal can be measured. Additionally, this method of using ThT dye has many advantages, such as low cost, simple design, ease of use, and speed. Moreover, the ThT neither changes the original properties of the aptamer nor affects the sensitivity of the aptamer. Hence, label-free detection using ThT dye is feasible. 

Here, we have isolated five novel aptameric ssDNA sequences that can specifically bind to fenitrothion. Further, we developed a fluorescent assay using 6-FAM labeling of three aptamers (FenA1, FenA2, and FenA5), which showed a higher affinity for fenitrothion. We report label-free detection of fenitrothion using an ssDNA aptamer (FenA2). Moreover, we tested the specificity of our sensor using non-target molecules, and the *K_d_* for each aptameric sensor was determined experimentally. The measured dissociation constants (*K_d_*s) are very low, indicating the aptamers have high specific binding to the insecticide fenitrothion. In the future, the assay be further be optimized for field sample analysis as a point of care diagnostic tool.

## 2. Results and Discussion

### 2.1. Selection of Fenitrothion-Specific ssDNA Aptamers Using SELEX-Based Strategy

**Figure 1:**
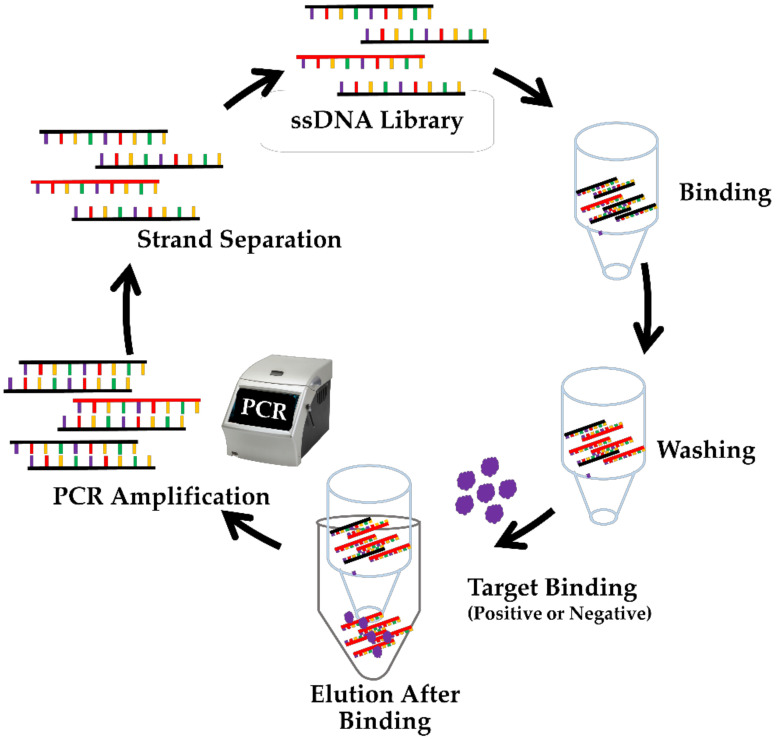
Illustrative scheme of the in vitro selection, SELEX process. The in vitro selection process begins with 1015 different ssDNA molecules and incubation with the target of interest, fenitrothion. Molecules that do not bind to fenitrothion are removed. Molecules that bind to fenitrothion are eluted, collected and amplified and reloaded after strand separation. It completes one round of an in vitro selection cycle.

The SELEX-method, which has proven to be suitable for identifying ssDNA aptamers specific to small molecules, was used in this study [24,25,34]. This approach is based on the properties of nucleic acids, which can change structure when they interact with the target to form the unique aptamer–target complex structure [35]. We employed the SELEX process in this study to identify ssDNA molecules specific to fenitrothion (Figure 1). In the SELEX process, the aptamers were subjected to complementary base-pairing with an oligonucleotide segment (an 18-mer capture strand), which carries a biotin-TEG at 3′-end. At the 3′-end of the capture strand, the biotin molecule binds to streptavidin resin with a strong affinity (protein–biotin interaction). The aptamer/capture strand complex is immobilized on the column containing streptavidin resin. Several washes with SELEX buffer (SB) were used to remove the unbound ssDNA from the streptavidin resin. After washing with a buffer, fenitrothion was added to the column. In the presence of fenitrothion, the aptamers that have an affinity for these small molecules will change the structure and separate from the capture strand. The ssDNA molecules bound to fenitrothion were collected and amplified using polymerase chain reactions (PCR).

### 2.2. Refinement of ssDNA Aptamers and Sequencing of Candidate Aptamers Obtained via SELEX

A total of 18 rounds of SELEX were carried out; the specific selection with 100 µM of fenitrothion was continued until round number 15. The counter selection was introduced post round 11 until the final round (Table 1). For each round, the eluted ssDNAs were taken to perform PCR, and PCR products were electrophoresed to check the profile of the binding ssDNA to the target (Figure S1). The list of oligonucleotide sequences used during the SELEX process is given in Table 2.

After round 15 of the SELEX process, ssDNAs from various selection concentrations were prepared for cloning and sequencing. A total of 24 plasmids were selected for sequencing. The sequence similarity was analyzed using the Omega web server [34]. The results obtained after sequencing in round 15 showed four candidate aptamers were found. Each aptamer had two copies accounting for 8%. A motif “GAGAGCC” was found in almost all sequences, whereas another motif, “CGCAGGGAGT”, was found in seven sequenced ssDNA candidates. A group including four sequences that differ only by a few nucleotides was also found (Table S1).

Sequencing results post round 18 at concentrations of 100 µM, 10µM, and 1 µM of fenitrothion showed that at a concentration of 100 µM of fenitrothion, a total of 11 candidate aptamers were found, of which two candidate aptamers had five copies, accounting for 20.8%, and one aptamer had two copies, accounting for 8% (Tables S1–S3). At a concentration of 10 µM, a total of 12 types of aptamer were found, of which five candidate aptamers were found and one candidate aptamer had four copies, accounting for 16%. Two other candidate aptamers had three copies, accounting for 12.5% and the other two had two copies making up 8% of the total (Tables S2 and S3). When 1 µm concentration of fenitrothion was applied, a total of 12 types of aptamers were found, of which three candidate aptamers were found (Table S4). One of them had three copies and accounted for 12.5%. Another had five copies (20.8%), and another has six copies (25%) (Figure S3). We found that two candidate aptamers appeared in all sequencing data with higher frequency, whereas three candidate aptamers also appeared in three out of four sequencing times with high frequency. The results suggest that the SELEX process had been tightly regulated and highly selective for our candidate aptamer identification.

### 2.3. Prediction of Secondary Structures of Selected ssDNA Aptamers 

**Figure 2:**
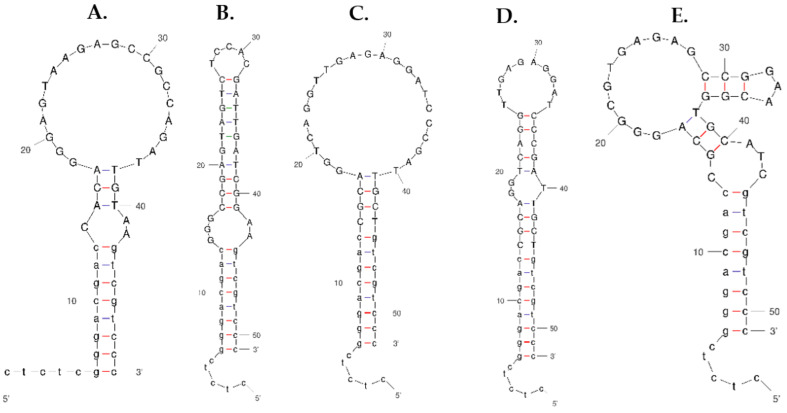
Secondary structures prediction of five candidate aptamers using Mfold web server [36]. (**A**) FenA, (**B**) FenA2, (**C**) FenA3, (**D**) FenA4, and (**E**) FenA5. Where Fen means fenitrothion, A means aptamer, and the number represents the number of the sequence.

A total of five candidate aptamers (Table 3) with a high number of copies were selected for secondary structure prediction by using the Mfold web server [36]. For 2D structure prediction, the conditions such as temperature, Na^+^, K^+^, and Mg^2+^ ion concentrations are set like in the SB (Figure 2). The predicted Gibb’s free energy value of these aptamers was −7.95 kcal/mol, −11.11 kcal/mol, −13.44 kcal/mol, −10.72 kcal/mol, and −10.62 kcal/mol for FenA1, FenA2, FenA3, FenA4, and FenA5, respectively.

### 2.4. Evaluation of Aptamer-FAM Fluorescence Quenching Efficiency

Among all sequences, five candidate aptamers were synthesized and chemically modified with an FAM molecule at the 5′ end called an FAM sensor (Table S5). This modification allows the sensor to exhibit fluorescence upon binding with target molecules. For determination of the binding efficiencies or affinities of the aptamers to fenitrothion, the *K_d_* of each candidate aptamer was calculated. To calculate *K_d_*, we followed a previously described method by Hu and Easley explained in equations 1, 2, and 3 [37]. Each aptamer displays two different binding affinities denoted as *Kd*,_eff1_, and *Kd*,_eff2_. *K_d_*_,eff1_ is the dissociation constant between the aptamer and quencher, which can be calculated using Equation (1):

*K_d_*_,eff1_ = [Aptamer][Quencher]/[Aptamer−Quencher] (1)

The *K_d_*_,eff2_ is a unitless constant measuring the equilibrium of the target-induced structure switching process, which can be calculated by Equation (2):

*K_d_*_,eff2_ = [Quencher][Aptamer−Target]/[Aptamer−Quencher][Target] (2)

Final *Kd* can then be calculated using Equation (3):

*K_d_* = *K_d_*_,eff1_/*K_d_*_,eff2_ (3)

**Figure 3:**
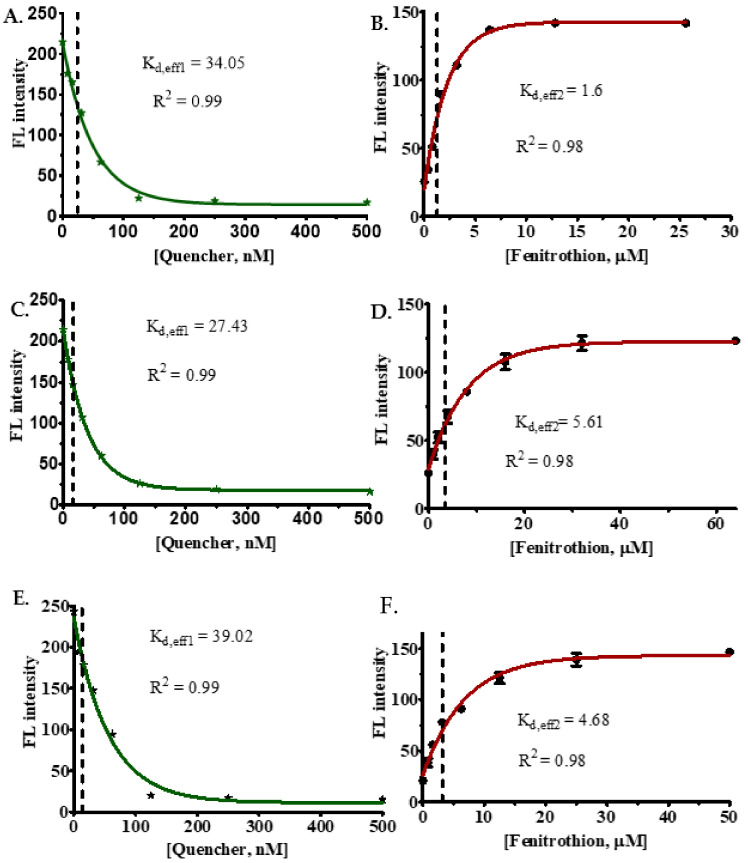
The experimental analysis of FAM sensors showing the quenching efficiency and measurement of *K_d_*_,eff1_ and *K_d_*_,eff2_ values for FenA1-FAM, FenA2-FAM, and FenA5-FAM sensors. (**A**) FenA1-FAM quenching; (**B**) FenA1-FAM sensor testing; (**C**) FenA2-FAM quenching; (**D**) FenA2-FAM sensor testing; (**E**) FenA5-FAM quenching; and (**F**) FenA5-FAM sensor testing. The dabcyl quencher was used in this assay (Figure S4).

To perform determination of *K_d_*_,eff1_, the concentration of aptamers was fixed at 50 nM, while eight different concentrations of quencher were applied (prepared using two times dilution series); as a result, the fluorescence intensity will be gradually decreased due to the quenching effect (Figure 3A,C,E). For the use of an aptamer in sensing, it should display 80% or higher quenching efficiency. In our analysis, two of the aptamers (FenA3-FAM and FenA4-FAM) did not produce sufficient quenching (Figure S2); hence they were not used for further experiments.

To evaluate *K_d_*_,eff2_, the aptamer–quencher complexes whose effective ratios were determined in the previous step were fixed, and eight different concentrations of the target were added. Since fenitrothion displays a higher affinity for the aptamer, its binding to aptamer causes structural-switching, which releases the quencher from the aptamer–quencher.

The statistical analysis was performed using a nonlinear regression curve fitting one phase decay using GraphPad Prism 5.0 software. The observed *K_d_*_,eff1_ of FenA1-FAM, FenA2-FAM, and FenA5-FAM were 34.05 nM, 27.43 nM, and 39.02 nM, respectively. These three aptasensors showed effective quenching at a ratio of 1:2.5 (1 sensor:2.5 quenchers). Next, to determine the binding affinity of FAM sensors with fenitrothion (i.e., *K_d_*_,eff2_), the complexes of each FAM-sensor and quencher were mixed at a ratio of 1:2.5, then different concentrations of fenitrothion were added, the fluorescence values were measured with excitation at 485 nm and collection of emission at 535 nm. Further, the changes in fluorescence signal were analyzed using a nonlinear regression analysis fitting one phase decay function of the GraphPad Prism 5.0 software, split into one phase decay (Figure 3B,D,F). *K_d,eff2_* is a unitless constant; *K_d_*_,eff2_ of sensors FenA1-FAM, FenA2-FAM, and FenA3-FAM are 1.6, 5.61, and 4.68, respectively. Eventually, the final *K_d_* values, which were obtained by applying Equation (3) for FenA1-FAM sensor, FenA2-FAM sensor, and FenA5-FAM sensor, were 21.28 nM, 4.889 nM, and 8.337 nM, respectively.

### 2.5. Evaluation of Target Specificity of Aptameric Sensors

**Figure 4:**
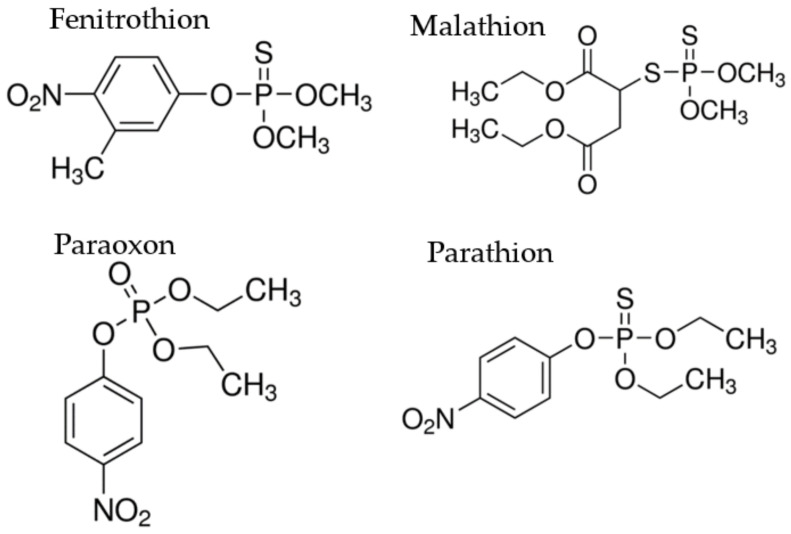
The chemical structure of fenitrothion and three other organophosphate insecticides; non-specific targets, namely malathion, paraoxon, and parathion, used to compare binding specificities of selected aptamers.

**Figure 5:**
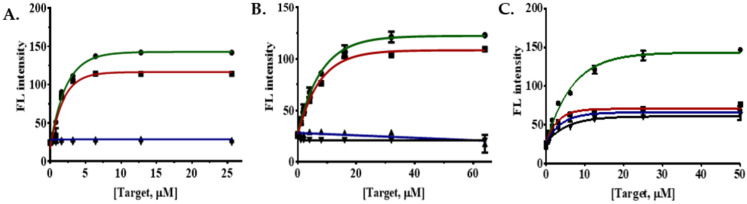
Experimental evaluation of the recognition specificity FAM-sensor: (**A**) FenA1-FAM, (**B**) FenA2-FAM, and (**C**) FenA5-FAM against its positive target —fenitrothion (green), and its negative targets—malathion (red), parathion (blue), and paraoxon (black).

The target specificity of the sensors is vital to obtain reliable and consistent results [25]. Hence, all three FAM-modified sensors, FenA1-FAM, FenA2-FAM, and FenA5-FAM, were subjected to cross-reactivity testing with three other organophosphate insecticides, namely malathion, paraoxon, and parathion, as negative targets (Figure 4). Although the FenA1-FAM (Figure 5A) and FenA2-FAM (Figure 5B) sensors were highly sensitive to fenitrothion binding, they also have relatively higher cross-reactivity with malathion. the FenA5-FAM sensor displayed very high specificity only for fenitrothion insecticide (Figure 5C).

### 2.6. Development of a Label-Free Method using ThT Displacement to Detect Fenitrothion

#### 2.6.1. ThT Displacement Assay

**Figure 6:**
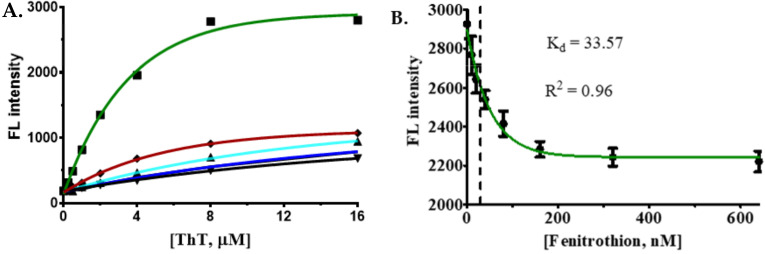
Evaluation of the binding potential of ssDNA aptamers with ThT. (**A**) Lighting up with ThT (green line represents FenA2; whereas the red, blue, aqua-blue, the black lines represent FenA1, FenA3, FenA4, and FenA5 ssDNA aptamers). Only FenA2 showed significant improvement in fluorescence upon binding with ThT, and other aptamers had much less affinity for ThT. (**B**) The FenA2-ThT sensor testing using different concentrations of fenitrothion. The *K_d_* obtained using this method was 33.57 nM (9.307 ppb) and LOD was 14.00 nM (3.881 ppb).

The development of label-free assays is highly desired for detecting small molecules [38,39]. Label-free detection provides several advantages over covalent modification of ssDNA aptamers with fluorophores. For instance, the fluorophore attachment interferes with the 3-D folding of the aptamer sequences and costs money to synthesize FAM-modified oligos [40]. Hence, to address the issues mentioned above, we developed a novel assay using Thioflavin T that does not require FAM-labeling [41,42]. Several reports suggest that aptamers with rich guanine nucleotides in their sequence can form duplex structures and possess an ability to bind with ThT. Such binding of ThT with G4-quadruplexes leads to an increase in ThT’s fluorescence intensity that can be recognized [43,44]; subsequently, the bound ThT can be displaced by a higher affinity target (fenitrothion) binding, which results in loss of fluorescence. This unique property of the “turn-on” and “turn-off” of fluorescence of ThT was exploited in this study to develop a biosensor to detect fenitrothion. This can be used to increase the sensitivity of the sensor. In this study, the candidate aptamers are modified, renamed, and called ThT sensors (Table S6), and all aptamers interacted with different concentrations of ThT. The results show that, among the candidate aptamers, only the FenA2 sensor has the ability to interact with ThT with peak fluorescence signal at 1:8 ratio (Sensor: ThT). Other sensors produced much lower fluorescence signals even at higher concentrations of ThT (Figure 6A).

When binding of ThT with each aptamer was tested, the FenA2 showed the highest affinity with ThT with an intense gain in fluorescence. Further, to verify the activity of the FenA2, the FenA2 and ThT were mixed at a ratio of 1:8 (Sensor: ThT). The fenitrothion binding displaces the ThT from the aptamer–ThT complex and reduces the fluorescence. The fluorescence was measured by exciting the solution at 425 nm and measuring the emission at 492 nm wavelength. The loss of fluorescence related to the concentration of fenitrothion was calculated using GraphPad Prism 5.0 software by a nonlinear regression curve fitting one phase decay model. The analysis revealed that the FenA2-ThT sensor was highly sensitive to fenitrothion with a *K_d_* 33.57 nM (9.307 ppb; Figure 6B) and LOD was 14.00 nM (3.881 ppb; Figure S3). We calculated the limit of detection (LOD) of our assay using the method that was described by Armbruster et al. (2008) [45].

#### 2.6.2. Recognition Specificity of FenA2-ThT Sensor

**Figure 7:**
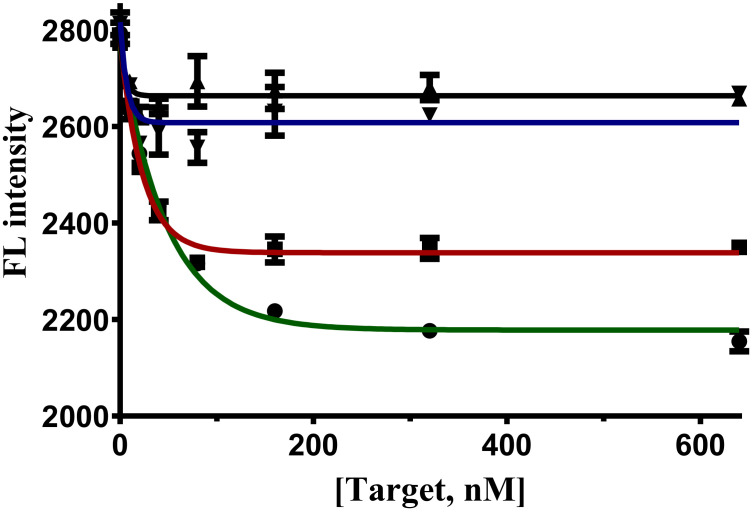
Recognition specificity of FenA2-ThT sensor against other organophosphate insecticides. The signal of FenA2-ThT in the presence of fenitrothion (green line with filled circles); in the presence of malathion (red line with squares); in the presence of parathion (blue line with inverted triangles); and in the presence of paraoxon (black lines with triangles).

Furthermore, to evaluate the specificity of the FenA2 sensor, the FenA2 sensor was reacted with negative targets. The results show that this sensor has high in selectivity for two negative targets, paraoxon and parathion. This sensor has a high cross-reactivity between fenitrothion and malathion (Figure 7). The FenA2-ThT bind to fenitrothion with high affinity. However, it can also bind to malathion with some specificity due to the closeness of the chemical structure.

#### 2.6.3. Label-Free Detection of Fenitrothion from Plant Tissue Extracts

**Figure 8:**
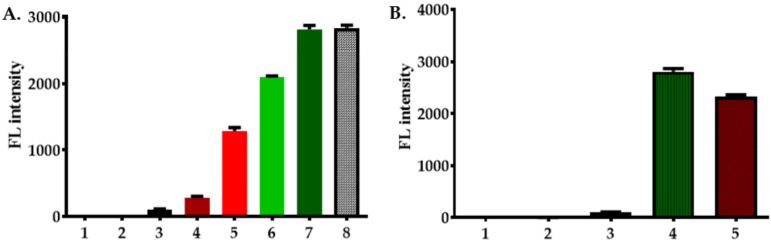
Label-free detection of fenitrothion using FenA2 sensor and ThT dye in plant tissue extracts. (**A**) Optimization of ThT fluorescence lighting: Measurement of ThT fluorescence upon binding with FenA2 aptamer using various dilutions of plant extracts. The fluorescence signal shown is of: (1) 1× buffer only; (2) 8 µM ThT dye only; (3) 1 µM FenA2 aptamer only; (4) 1 µM FenA2: 8 µM ThT in the 1× plant extract dilution; (5) 1 µM FenA2: 8 µM ThT in the 10× plant extracted dilution; (6) 1 µM FenA2: 8 µM ThT in the 100× plant extracted dilution; (7) 1 µM FenA2: 8 µM ThT in the 1000× plant extracted dilution; (8) 1 µM sensor: 8 µM ThT in 1× SB buffer. (**B**) FenA2 Sensor testing for analysis of fenitrothion in plant extract. The fluorescence signal shown is of: (1) 1× buffer only; (2) 8 µM ThT; (3) 1 µM FenA2 Aptamer only; (4) 1 µM FenA2: 8 µM ThT in the 1000× plant extract dilution; and (5) 1 µM FenA2: 8 µM ThT with 50 nM fenitrothion spiked in the 1000× plant extract dilution.

Chinese cabbage plant leaves were used for label-free detection of fenitrothion using the FenA2 aptamer sensor and ThT dye. The plant tissue extracts were prepared following the aqueous extraction method in 1x SB buffer. The tissue extract was subjected to serial dilution for ThT detection. Dilution of plant extract is important to reduce the autofluorescence of the plant leaves and reduce the acetone content in the extract; hence various dilutions were used to obtain an optimal signal (Figure 8A). We found that 1000× dilution of plant extract yielded the highest fluorescence signal. After optimization of the dilution, the plant extracts were spiked with fenitrothion insecticide at 50 nM concentration, and these spiked samples were analyzed using the novel aptamer FenA2 developed in this study. The ThT-based assay could successfully detect the presence of fenitrothion (Figure 8B).

Overall, we have developed a novel application of ssDNA aptamers for the detection of the toxic insecticide fenitrothion from plant tissue extracts. Here, we identified five novel ssDNA aptamers that can specifically bind to fenitrothion. Of these, three aptamers (FenA1-FAM, FenA2-FAM, and FenA5-FAM) were modified with 6-FAM and used for its detection. Moreover, one aptamer (FenA2) was found to bind with ThT dye. Further, using FenA2, we developed a label-free assay for the detection of fenitrothion with a very low *K_d_* value. All aptamers displayed a very low *K_d_* value (in nanomolar or ppb range). The sensor could be further developed to integrate real-sample testing in field conditions.

## 3. Materials and Methods

### 3.1. DNA Oligonucleotides snd Chemical Reagents

In this study, a library of ssDNA and all oligonucleotides was purchased from COSMO Genentech Company (Seoul, South Korea). Fenitrothion, malathion, paraoxon, and parathion were purchased from Kemidas Company (Suwon-si, Gyeonggi-do, South Korea). Other chemicals such as Tris-HCl, HCl, NaOH, NaCl, KCl, HEPES, TBE, TAE, MgCl_2_, Thioflavin T (ThT), and agarose powder of molecular biology grade were purchased from Sigma-Aldrich Co, Ltd. (South Korea). All reactions were performed using deionized double distilled water (ddH_2_O). Fenitrothion and negative targets, all hydrophobic compounds; were first dissolved in acetone and then were diluted to the working concentration with 1 × SB (SELEX buffer) using serial or intermediate dilutions to ensure solubility.

### 3.2. Reagents and Buffers for SELEX

In this study, three different types of buffer were used to maintain DNA stability and integrity. First, the SELEX buffer (SB) was used with the following components: 1 × SB (20 mM HEPES, 1 M NaCl, 10 mM MgCl_2_, 5 mM KCl, pH 7.5). SB was used during the SELEX procedure. The second buffer, separation buffer (SEPB), has the following components: (20 mM HEPES, 300 mM NaCl, pH 7.5). This buffer was used to separate double-stranded DNA fragments obtained from the PCR reaction. Finally, the ThT buffer (TB) had the following components: (10mM Tris-HCl and 10mM NaCl at pH 7.0). This buffer was used to perform binding experiments with ThT.

### 3.3. Selection of Desired Candidate ssDNA Using SELEX Process

The selection process we employed is based on Yang et al.’s (2016) protocol [24]. For the first round, 500 pmoles of the ssDNA library were mixed with 2500 pmoles of capture strand in the 250 µL of 1 × SB. The mixture was heated to 95 °C for 5 min. The solution was kept at room temperature for 15 min. A mini-BioRad column was prepared after washing with 300 µL 1 × SB to remove air bubbles, then 250 µL streptavidin resin was added to the column. Then, streptavidin resin was washed six times with 1 × SB to neutralize the pH. The library capture-strand complex was passed through the column three times and unbound ssDNAs with streptavidin resin were collected. Then, the column was washed at least ten times with 1 × SB, 250 µL each time, to collect the elution. Additionally, the column was eluted three times with negative targets. This was followed by ten washes with SB buffer, then 250 µL of 100 µM positive targets, fenitrothion were used to get the elutions. All elutions are used to check binding using the PCR reaction.

The PCR conditions were as follows: one cycle denaturation at 95 °C for 2 min, N cycles of [denaturation for 15 sec at 92 °C; followed by annealing at 59 °C for 30 s; then extension at 72 °C for 45 s], and final cycle of extension for 2 min at 72 °C. The PCR products were separated using electrophoresis using 100 V for 20 min in 3% agarose gel with 1X TAE buffer. The electrophoresed products were stained with ethidium bromide (EtBr) and then the bands under the UV light of the gel were recorded.

For preparing a new library for the next round, 1 mL of PCR sample reaction was prepared, the same components as in the small-scale PCR, but biotinylated reverse primer was used here. A total of 100 µL of PCR sample was taken out of the device to 5 PCR tubes and run at different cycles to estimate the number of PCR cycles needed to apply to a large PCR (normally 9 + 2 cycles are applied first). PCR products were concentrated to 100 µL using 3 KDa Amicon^®^ Ultra and centrifuged at 19,320× *g*. A new column was prepared, where 250 µL streptavidin resin was added to the column and the beads were washed six times with 1× separation buffer (SEPB). Then, concentrated PCR products were added to the column. The elute was collected and reapplied to the column 3 times. After that, the column was washed ten times with 1 × SEPB. A stopper was used to close the column; 400 µL of 0.2 M NaOH was added to the column and incubated for 10 min at room temperature. The eluted fraction was collected, an equal volume of 0.2 M HCl was added to adjust the pH to 7.5, and then 750 µL of 2 × SB was added. The solution was concentrated to 100 µL by using a 3 KDa Amicon^®^ Ultra and centrifuged at 19,320× *g*.

### 3.4. Cloning and Sequencing of Selected Candidate ssDNA Aptamers

The enriched DNA library obtained post round 15 and round 18 were amplified. The PCR amplification is carried out as follows: one cycle of denaturation at 95 °C for 2 min, then nine cycles of 92 °C for 15 s, annealing at 59 °C 30 s, extension at 72 °C for 45 s, and finally 1 cycle of 72 °C for 2 min. The non-biotinylated reverse primers were used in this step. Cloning was performed as per the manufacturer’s instructions. T-blunt vector kit was used to clone the fragments, and plasmids were extracted for sequencing by COSMO Genentech company.

### 3.5. Bioinformatic Analysis to Study Sequence Similarity and Predict The Secondary Structure of The Candidate Aptamers

All the sequenced clones were analyzed for the sequence similarity of candidate aptamers using the Clustal Omega web-server [33]. Next, we used the Mfold web-server to predict the structure of candidate aptamers [25].

### 3.6. Measurement of Binding Affinity

#### 3.6.1. The FAM Assays

To determine the *K_d_* of each of the candidate aptamers (Table S7), we performed the following steps: first, we identified the *K_d_*_,eff1_ using a fixed concentration of 2 × FAM-Sensor (100 nM) and eight different concentrations of quencher were prepared from 0 to 1000 nM (1000, 500, 250, 125, 62.5, 31.25, 15.625, 0 nM) by two times serial dilution. A total of 60 µL of 2× FAM sensor and 60 µL of 2× of each concentration quencher were mixed and treated at 95 °C for 5 min and were cooled down slowly to room temperature in a dark room for ~20 min. Then, 100 µL of each mixture was transferred into a 96-well microplate (non-binding surface, flat bottom; black polystyrene assay plates; Corning, Corning, NY, USA). The fluorescence intensity of each well was recorded on a Gemini XPS Spectra Max Multi-mode microplate reader (Molecular Devices, San Jose, CA, USA). The samples were excited by a laser at 485 nm and the emission spectra were recorded at 535 nm. Using nonlinear least-squares progression (GraphPad Prism 5.0 software), the binding affinity curve was plotted to generate *K_d_*_,eff1_.

For calculation of *K_d_*_,eff2_, the final concentration of both sensor and quencher were kept at the molar ratio where quenching was > 80% in the previous experiment conducted to calculate *K_d_*_,eff1_ (i.e., 1 sensor: 5 quenchers); and the concentration of fenitrothion was changed. The mixture of sensor and quencher was heated to 95 °C for 5 min for denaturation of aptamers and were cooled down to room temperature for 30 min. An equal volume of fenitrothion (diluted in SB) was added and incubated at room temperature for 40 min to allow the binding with aptamer and release of quencher. A total of 100 µL of each mixture was transferred into wells (96-well microplate) and the fluorescence spectra were acquired by excitation at 485 nm and emission at 535 nm. All measurements were made in three technical replicates. The observed fluorescence intensities were then plotted using nonlinear least-squares fitting (GraphPad Prism 5.0 software).

#### 3.6.2. Label-Free Detection of Fenitrothion using ThT Dye Displacement

For the analysis of *K_d_* using ThT dye (Figure S4) displacement assay, the concentration of aptamer was fixed at 1 µM, and a varied concentration of ThT was applied. Twice the concentration of the aptamer was denatured at 95 °C in 5 min and then cooled down to room temperature for 30 min. A total of 60 µL of 2× concentration of the aptamers and 60 µL of 2× concentration of ThT were mixed and allowed to interact by incubation at 30 °C for 30 min. Finally, 100 µL of each reaction mixture was added to 96-well microplates, and the fluorescence was measured by excitation and emission at 425 nm and 492 nm, respectively. The data were analyzed using GraphPad Prism 5 software and plotted with the nonlinear regression function.

Next, the analysis of fenitrothion binding with the aptamers was performed using 2× of the Sensor/ThT complex; 60 µL of 2× of the Sensor/ThT complex was incubated at 30 °C for 30 min. Then, the same volume of each concentration from 0 to 600 nm of fenitrothion was added, and the binding of fenitrothion or displacement of ThT dye was observed after incubation at 30 °C for more than 30 min. A total of 100 µL of each reaction was added to a 96-well black flat bottom microplate and fluorescence was measured by excitation and emission at 425 nm and 492 nm, respectively. GraphPad Prism 5.0 software was used to analyze the data, and the curve was plotted using the nonlinear regression function fitting one phase decay.

#### 3.6.3. Measurement of Specificity of Both Assays

For measuring the target specificity of the 6-FAM quenching assay and ThT displacement assay, the aptamers were reacted with non-specific (negative) targets; the small molecule insecticides like malathion, paraoxon, and parathion, which are structurally similar to fenitrothion. The binding curves are plotted using nonlinear least-squares fitting using GraphPad Prism 5.0 software.

### 3.7. Detection of Fenitrothion from Plant Extract

A total of 50 g of Chinese cabbage plant tissue was ground and dissolved in 50 mL of 2× SB buffer, and the mixture was filtered to collect the tissue extract. Further, the extract was centrifuged at 19,320× g for 10 min. Upon centrifugation, the debris were removed, and the supernatant was collected for analysis. The supernatant was diluted 10 times by serial dilution with 1× SB buffer. The tissue extract was spiked with fenitrothion by adding 50 nM as the final concentration. The presence of fenitrothion was detected as described earlier in the methods section.

## 4. Conclusions

In conclusion, we have successfully developed a novel aptamer-based biosensing platform to detect fenitrothion with high affinity and high specificity. Furthermore, we demonstrated a label-free detection method using ThT dye displacement for the insecticide. To the best of our knowledge this is the first label-free ssDNA aptamer sensor that binds to fenitrothion. With the development of such high-quality aptamers, we are hopeful of developing efficient tests in the future that can be used to detect fenitrothion contamination in nature.

## Figures and Tables

**Table 1:** The concentrations of specific and non-specific targets used during the SELEX process.

Round	Positive Selection	Negative Selection
1 to 10	100 µM of fenitrothion	-
11 and 15	100 µM of fenitrothion	1 malathion µM, 1 µM of paraoxon, 1 µM of parathion
16 to 18	1 µM, 10 µM, 100 µM of fenitrothion	1 malathion µM, 1 µM of paraoxon, 1 µM of parathion

(-) negative selection is not applied in these rounds.

**Table 2:** The list of all DNA molecules used in the SELEX process.

Name	Sequence
ssDNA Library	5′- GGAGGCTCTCGGGACGAC(N30)GTCGTCCCGCCTTTAGGATTTACAG-3′
Capture strand	5′- GTCGTCCCGAGAGCCATA-Biotin-TEG-3′
Forward Primer	5′-GGAGGCTCTCGGGACGAC-3′
Reverse Primer	5′-CTGTAAATCCTAAAGGCGGGACGAC-3′
Biotinylated-Reverse Primer	5′-Biotin-CTGTAAATCCTAAAG GCGGGACGAC-3′

**Table 3:** The list of candidate aptamers used in this study. The top five sequences with a high number of copies after sequencing are listed here.

Name	Sequence (5′ to 3′)	Number of Copies
FenA1	ctctcgggacgac CACAGGGAGTAAGAGGCCGCCAGATTGTAA gtcgtccc	15
FenA2	ctctcgggacgac GGGCCGAGTAGTCTCCACGATTGATCGGAA gtcgtccc	14
FenA3	ctctcgggacgac CGCAGGTCAGGTTGAGAGGATCCCGATTGCT gtcgtccc	8
FenA4	ctctcgggacgac CGCAGGTTGTCTGAGCCGACAGGTTGCAT gtcgtccc	5
FenA5	ctctcgggacgac CGCAGGGCGTGAGAGCCGGAACGGTGCATC gtcgtccc	5

Partial primer regions are denoted by small letters. The capital/center letters are the aptamer region.

## Data Availability

The data presented in this study are available on request from the corresponding author.

## Supplementary Materials

The following are available online at https://www.mdpi.com/article/10.3390/ijms221910846/s1: Figure S1. Agarose gel electrophoresis of candidate ssDNA aptamers post SELEX process; Figure S2. Quenching efficiency assay for FenA3-FAM sensor and FenA4-FAM sensor; Figure S3. Calculation of LOD using ThT assay and FenA2 aptamer. Calculation of LOD using ThT assay and FenA2 aptamer; Table S1. Sequencing data post round 15 of SELEX selection; Table S2. Sequencing data post round 18 at 100 µM of fenitrothion application; Table S3. Sequencing data post round 18 at 10 µM of fenitrothion application; Table S4. Sequencing data post round 18 at 1 µM of fenitrothion application; Table S5. List of novel FAM sensors designed in this study; Table S6. List of novel ssDNA aptamers used for ThT sensor design; and Table S7. The list of all DNA molecules used in the SELEX process.

## Abbreviations

MRE	Molecular Recognition Element
BSA	Bovine Serum Albumin
LOD	Limit of Detection
ssDNA	Single-stranded DNA
FAM	6-Carboxyfluorescein
ThT	Thioflavin T
6-FAM	6-Fluorescein
USEPA	United States Environmental Protection Agency
SELEX	Systemic Evolution of Ligand by Exponential Enrichment
WHO	World Health Organization
FAO	Food and Agriculture Organization
Biotin-TEG	Biotin-Tetraethylene Glycol
