# Supplementary material for: Novel DNA Aptameric Sensors to Detect the Toxic Insecticide Fenitrothion
Source: Int J Mol Sci. 2021 Oct 7;22(19):10846. doi: 10.3390/ijms221910846 (PMC8509669; doi:10.3390/ijms221910846)
Supplement: Supplementary file 1 [file ijms-22-10846-s001.zip › ijms-1390781-supplementary.pdf]

Supplementary Information

Novel DNA Aptameric Sensors to Detect the Toxic Insecticide Fenitrothion

Kien Hong Trinh[1,2,‡], Ulhas Sopanrao Kadam[1,‡], Jinnan Song[1], Yuhan Cho[1], Chang Ho Kang[1], Kyun Oh Lee[1], Chae Oh Lim[1], Woo Sik Chung[1], Jong Chan Hong[1*]

[1] Division of Life Science and Applied Life Science, Plant Molecular Biology and Biotechnology Research Center, Gyeongsang National University, Jinju, Gyeongnam, 52828, Republic of Korea.

2 Faculty of Biotechnology, Vietnam National University of Agriculture, Hanoi City, Vietnam.

[*]Correspondence: jchong@gnu.ac.kr

[‡]These authors contributed equally as co-first authors to this work.

Figure Legends..

Figure S1. Agarose gel electrophoresis of candidate ssDNA aptamers post SELEX process.

Figure S2. Quenching efficiency assay for FenA3-FAM sensor and FenA4-FAM sensor.

Figure S3. Calculation of LOD using ThT assay and FenA2 aptamer.

Table Legends..

Table S1. Sequencing data post round 15 of SELEX selection.

Table S2. Sequencing data post round 18 at 100 μM of fenitrothion application.

Table S3. Sequencing data post round 18 at 10 μM of fenitrothion application.

Table S4. Sequencing data post round 18 at 1 μM of fenitrothion application.

Table S5. List of novel FAM sensors designed in this study.

Table S6. List of novel ssDNA aptamers used for ThT sensor design.

Table S7. The list of all DNA molecules used in the SELEX process..

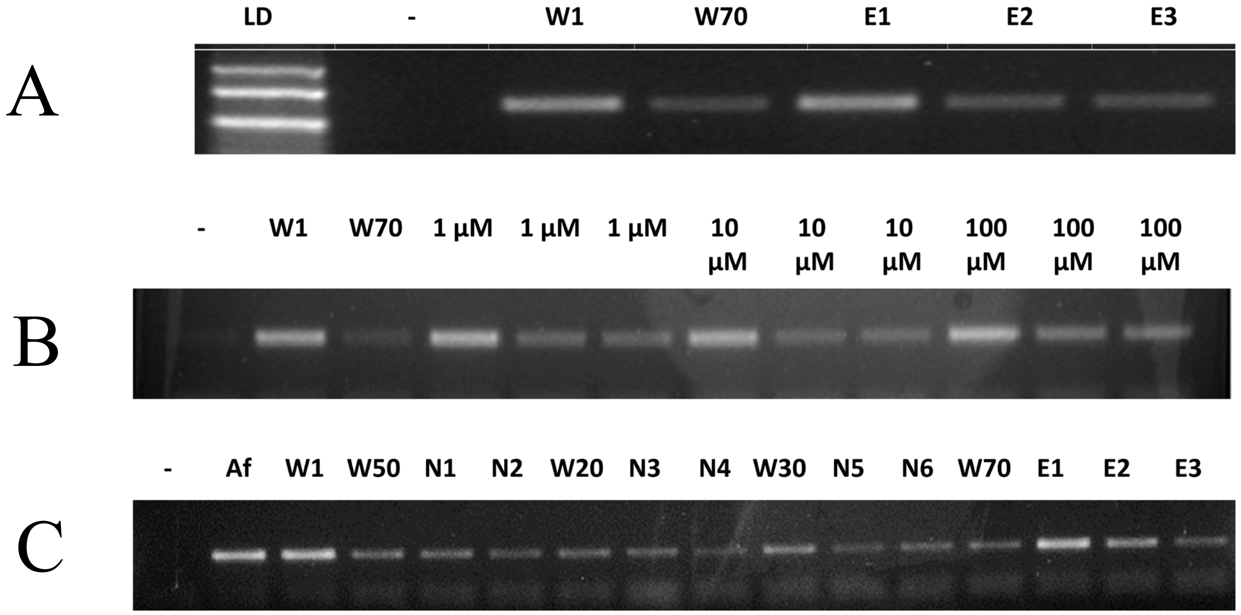

Figure S1. Gel electrophoresis of candidate ssDNA aptamer post SELEX rounds. A) Electrophoresis image post round number 15; B) Electrophoresis image post round number 16; C) Electrophoresis image post round number 18. The abbreviations are as: LD means DNA ladder; – means negative control; W1 means first wash with 1x SB; W70 mean wash final time with 1x SB; E1/E2/E3 mean eluted fraction after fenitrothion treatment for positive selection; 1µM, 10 µM, 100 µM mean respective concentrations of fenitrothion were applied; Af mean after column W50; N1/N2 mean when paraoxon was applied as a negative target during negative selection; N3/N4 mean when parathion was applied as a negative target in the negative selection and N5/N6 mean when malathion was applied as a negative target in the negative selection.

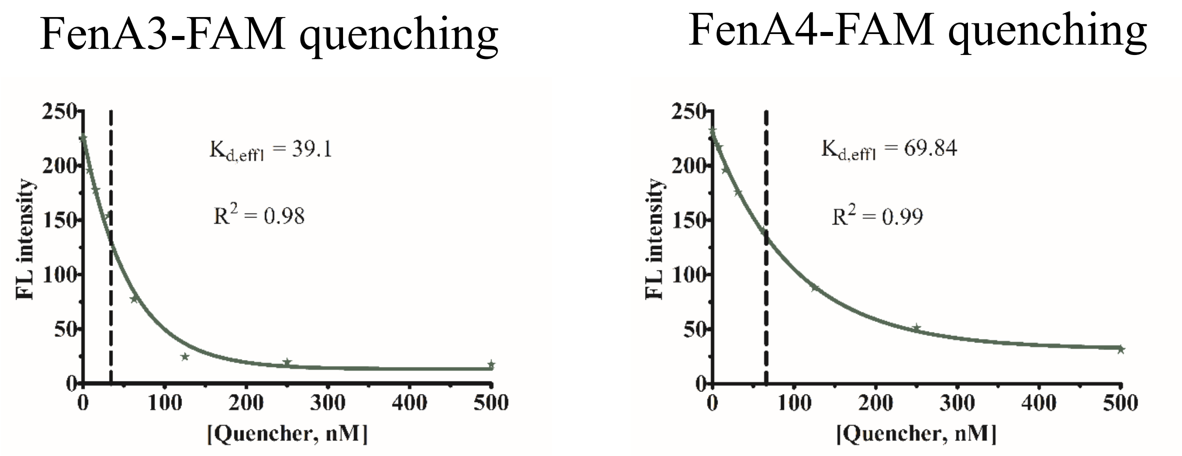

Figure S2. Quenching efficiency assay for FenA3-FAM sensor and FenA4-FAM sensor

Figure S3. Calculation of LOD using ThT assay and FenA2 aptamer

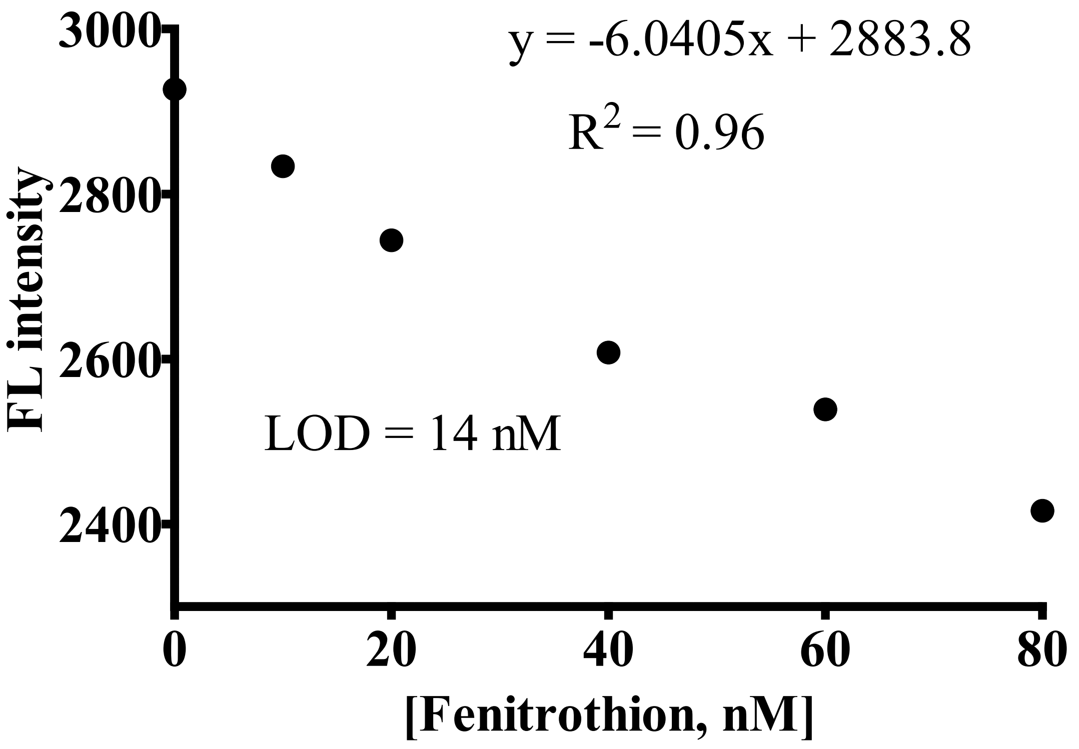

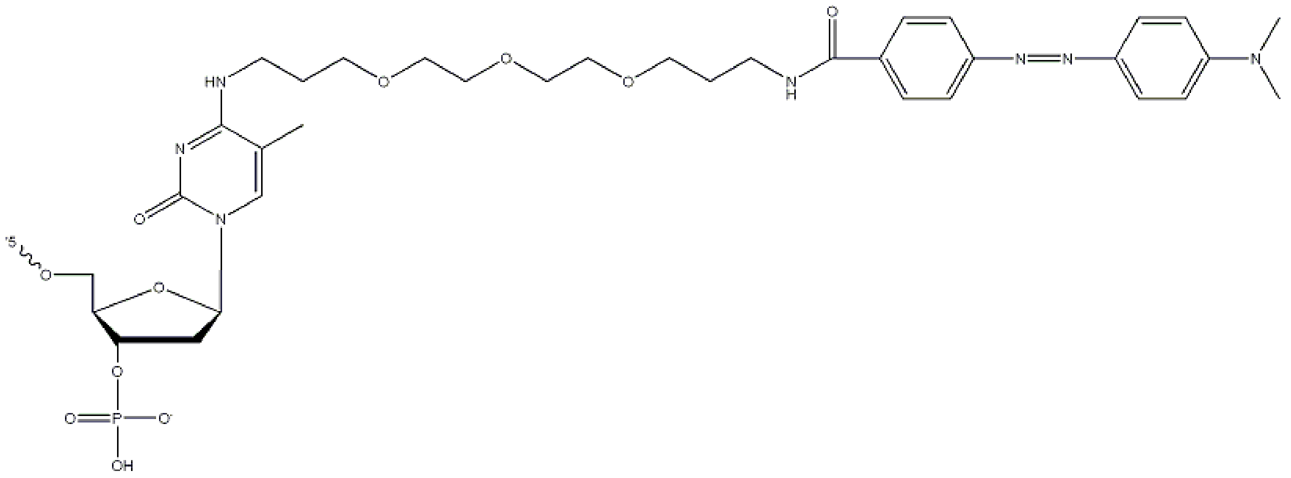

Figure S4. Chemical structure of dabcyl quencher used in the assay (Source: https://sg.idtdna.com/site/Catalog/Modifications/Product/1067).

Table S1: Sequencing result at post round 15

Name of clone	Forward primer	Sequence (5′→3')	Reverse primer
Fen100-11	ctctcgggacgac	GGGCCGAGTGTCTCCTTTCGATGGAATTGCAT	gtcgtccc
Fen100-23	ctctcgggacgac	ACGCTACACTCCTGGAGCCTGGCCGACCGTTGT	gtcgtccc
Fen100-24	ctctcgggacgac	ACGATGCACCGTTCCGGCTCTCACGCCCTGCGT	gtcgtccc
Fen100-7	ctctcgggacgac	GAGGGCTGGAATTGTATGAAGGCGTGGTATGT	gtcgtccc
Fen100-20	ctctcgggacgac	CTAGGGTTCATTGCGTGCGTGAGTTTACGAGT	gtcgtccc
Fen100-5	ctctcgggacgac	GATGTTAGGGTTCTTTGCGTGAGTTTACGAGT	gtcgtccc
Fen100-18	ctctcgggacgac	CGCAGGTCAGGTTGAGAGGATCCCGATTGCTGT	gtcgtccc
Fen100-22	ctctcgggacgac	CGCAGGTCAGGTTGAGAGGATCCCGATTGCTGT	gtcgtccc
Fen100-17	ctctcgggacgac	CGTAGGGAGTAGTTGGAGCCGCCTGATGCAGT	gtcgtccc
Fen100-6	ctctcgggacgac	CGCAGGGAGTGGAACCCAATTAGGTGCGTGGT	gtcgtccc
Fen100-9	ctctcgggacgac	CGCAGGGAGTGGAGCCGAATTAGGTTGGAGGT	gtcgtccc
Fen100-15	ctctcgggacgac	CGCAGGGAGTGAGAGCTGGAAGGATTGGTCGT	gtcgtccc
Fen100-2	ctctcgggacgac	CGCAGGGCGTGAGAGCCGGAACGGTGCATCGT	gtcgtccc
Fen100-1	ctctcgggacgac	CGCAGGTTGTGAGAGCCGACAGGTTGCATCGT	gtcgtccc
Fen100-16	ctctcgggacgac	CGCAGGTTGTGAGAGCCGACAGGTTGCATCGT	gtcgtccc
Fen100-8	ctctcgggacgac	CCGTGCCAAAGAGAGCCGAACAGATGCATAAGT	gtcgtccc
Fen100-19	ctctcgggacgac	CCGTGCCAAAGAGAGCCGAACAGATGCATAAGT	gtcgtccc
Fen100-12	ctctcgggacgac	CAAAGGGAGAGAGAGCTGGGGGATTGTTGTGT	gtcgtccc
Fen100-3	ctctcgggacgac	CACAGGGAGTAAGAGGCCGCCAGATTGTAAGT	gtcgtccc
Fen100-13	ctctcgggacgac	CACAGGGAGTAAGAGGCCGCCAGATTGTAAGT	gtcgtccc
Fen100-4	ctctcgggacgac	GCAGAAGTAGAGAGCCGGTACCATGGCAGTGT	gtcgtccc
Fen100-14	ctctcgggacgac	GGAAGGAGTTAAAGAGCCGGAACAGTTGCAGT	gtcgtccc
Fen100-10	ctctcgggacgac	GGGGAAAGTGCCGTGAGACTCCTAGTTGCAGT	gtcgtccc
Fen100-21	ctctcgggacgac	GGAGTGAGGGCCGCAAAGGGGGAGGGATGGGT	gtcgtccc

The highlighted sequences represent candidate aptamers.

Table S2: Sequencing result at post round 18 at 100 μM of Fenitrothion application.

Name of clone	Fw primer	Sequence (5'◎ 3')	Rev primer
FenA100-3	ctctcgggacgac	GGGCCGAGTAGTCTCCACGATTGATCGGAAT	gtcgtccc
FenA100-9	ctctcgggacgac	GGGCCGAGTAGTCTCCACGATTGATCGGAAT	gtcgtccc
FenA100-14	ctctcgggacgac	GGGCCGAGTAGTCTCCACGATTGATCGGAAT	gtcgtccc
FenA100-18	ctctcgggacgac	GGGCCGAGTAGTCTCCACGATTGATCGGAAT	gtcgtccc
FenA100-20	ctctcgggacgac	GGGCCGAGTAGTCTCCACGATTGATCGGAAT	gtcgtccc
FenA100-4	ctctcgggacgac	CACAGGGAGTAAGAGGCCGCCAGATTGTAAGT	gtcgtccc
FenA100-7	ctctcgggacgac	CACAGGGAGTAAGAGGCCGCCAGATTGTAAGT	gtcgtccc
FenA100-13	ctctcgggacgac	CACAGGGAGTAAGAGGCCGCCAGATTGTAAGT	gtcgtccc
FenA100-21	ctctcgggacgac	CACAGGGAGTAAGAGGCCGCCAGATTGTAAGT	gtcgtccc
FenA100-22	ctctcgggacgac	CACAGGGAGTAAGAGGCCGCCAGATTGTAAGT	gtcgtccc
FenA100-6	ctctcgggacgac	CGCAGGTCAGGTTGAGAGGATCCCGATTGCTGT	gtcgtccc
FenA100-15	ctctcgggacgac	CGCAGGTCAGGTTGAGAGGATCCCGATTGCTGT	gtcgtccc
FenA100-8	ctctcgggacgac	CCGTAACGTGAGACTCCTAGGATTGCGAACGT	gtcgtccc
FenA100-24	ctctcgggacgac	CACGGCATACGAGAGGCTCCGATGATGTTGCAT	gtcgtccc
FenA100-5	ctctcgggacgac	CAACGGGAGTCATGAGGCTGATAGATTAAGCGT	gtcgtccc
FenA100-17	ctctcgggacgac	GGGAAGTGAGAGCCGAGTGTAGAGCAGCAAGT	gtcgtccc
FenA100-16	ctctcgggacgac	CGCAGGGCGTGAGAGCCGGAACGGTGCATCGT	gtcgtccc
FenA100-2	ctctcgggacgac	GGGGGAGAGAGAGCCGGAATGATTGCATGATGT	gtcgtccc
FenA100-12	ctctcgggacgac	CGTAGGGAGTCAGAGCTGGAAGGATTGGTAGT	gtcgtccc
FenA100-1	ctctcgggacgac	CGGTGAAAAGGACGGAATTCCTTTGCAACTAGT	gtcgtccc

The highlighted sequences represent candidate aptamers.

Table S3: sequencing result at post round 18 at 10 μM of Fenitrothion application.

Name of clone	Fw primer	Sequence (5'◎ 3')	Rev primer
FenA100-3	ctctcgggacgac	GGGCCGAGTAGTCTCCACGATTGATCGGAAT	gtcgtccc
FenA100-9	ctctcgggacgac	GGGCCGAGTAGTCTCCACGATTGATCGGAAT	gtcgtccc
FenA100-14	ctctcgggacgac	GGGCCGAGTAGTCTCCACGATTGATCGGAAT	gtcgtccc
FenA100-18	ctctcgggacgac	GGGCCGAGTAGTCTCCACGATTGATCGGAAT	gtcgtccc
FenA100-20	ctctcgggacgac	GGGCCGAGTAGTCTCCACGATTGATCGGAAT	gtcgtccc
FenA100-4	ctctcgggacgac	CACAGGGAGTAAGAGGCCGCCAGATTGTAAGT	gtcgtccc
FenA100-7	ctctcgggacgac	CACAGGGAGTAAGAGGCCGCCAGATTGTAAGT	gtcgtccc
FenA100-13	ctctcgggacgac	CACAGGGAGTAAGAGGCCGCCAGATTGTAAGT	gtcgtccc
FenA100-21	ctctcgggacgac	CACAGGGAGTAAGAGGCCGCCAGATTGTAAGT	gtcgtccc
FenA100-22	ctctcgggacgac	CACAGGGAGTAAGAGGCCGCCAGATTGTAAGT	gtcgtccc
FenA100-6	ctctcgggacgac	CGCAGGTCAGGTTGAGAGGATCCCGATTGCTGT	gtcgtccc
FenA100-15	ctctcgggacgac	CGCAGGTCAGGTTGAGAGGATCCCGATTGCTGT	gtcgtccc
FenA100-8	ctctcgggacgac	CCGTAACGTGAGACTCCTAGGATTGCGAACGT	gtcgtccc
FenA100-24	ctctcgggacgac	CACGGCATACGAGAGGCTCCGATGATGTTGCAT	gtcgtccc
FenA100-5	ctctcgggacgac	CAACGGGAGTCATGAGGCTGATAGATTAAGCGT	gtcgtccc
FenA100-17	ctctcgggacgac	GGGAAGTGAGAGCCGAGTGTAGAGCAGCAAGT	gtcgtccc
FenA100-16	ctctcgggacgac	CGCAGGGCGTGAGAGCCGGAACGGTGCATCGT	gtcgtccc
FenA100-2	ctctcgggacgac	GGGGGAGAGAGAGCCGGAATGATTGCATGATGT	gtcgtccc
FenA100-12	ctctcgggacgac	CGTAGGGAGTCAGAGCTGGAAGGATTGGTAGT	gtcgtccc
FenA100-1	ctctcgggacgac	CGGTGAAAAGGACGGAATTCCTTTGCAACTAGT	gtcgtccc

The highlighted sequences represent candidate aptamers.

Table S4: sequencing result at post round 18 at 1μM of Fenitrothion application.

Name of clone	Fw primer	Sequence (5'◎ 3')	Rev primer
FenA100-3	ctctcgggacgac	GGGCCGAGTAGTCTCCACGATTGATCGGAAT	gtcgtccc
FenA100-9	ctctcgggacgac	GGGCCGAGTAGTCTCCACGATTGATCGGAAT	gtcgtccc
FenA100-14	ctctcgggacgac	GGGCCGAGTAGTCTCCACGATTGATCGGAAT	gtcgtccc
FenA100-18	ctctcgggacgac	GGGCCGAGTAGTCTCCACGATTGATCGGAAT	gtcgtccc
FenA100-20	ctctcgggacgac	GGGCCGAGTAGTCTCCACGATTGATCGGAAT	gtcgtccc
FenA100-4	ctctcgggacgac	CACAGGGAGTAAGAGGCCGCCAGATTGTAAGT	gtcgtccc
FenA100-7	ctctcgggacgac	CACAGGGAGTAAGAGGCCGCCAGATTGTAAGT	gtcgtccc
FenA100-13	ctctcgggacgac	CACAGGGAGTAAGAGGCCGCCAGATTGTAAGT	gtcgtccc
FenA100-21	ctctcgggacgac	CACAGGGAGTAAGAGGCCGCCAGATTGTAAGT	gtcgtccc
FenA100-22	ctctcgggacgac	CACAGGGAGTAAGAGGCCGCCAGATTGTAAGT	gtcgtccc
FenA100-6	ctctcgggacgac	CGCAGGTCAGGTTGAGAGGATCCCGATTGCTGT	gtcgtccc
FenA100-15	ctctcgggacgac	CGCAGGTCAGGTTGAGAGGATCCCGATTGCTGT	gtcgtccc
FenA100-8	ctctcgggacgac	CCGTAACGTGAGACTCCTAGGATTGCGAACGT	gtcgtccc
FenA100-24	ctctcgggacgac	CACGGCATACGAGAGGCTCCGATGATGTTGCAT	gtcgtccc
FenA100-5	ctctcgggacgac	CAACGGGAGTCATGAGGCTGATAGATTAAGCGT	gtcgtccc
FenA100-17	ctctcgggacgac	GGGAAGTGAGAGCCGAGTGTAGAGCAGCAAGT	gtcgtccc
FenA100-16	ctctcgggacgac	CGCAGGGCGTGAGAGCCGGAACGGTGCATCGT	gtcgtccc
FenA100-2	ctctcgggacgac	GGGGGAGAGAGAGCCGGAATGATTGCATGATGT	gtcgtccc
FenA100-12	ctctcgggacgac	CGTAGGGAGTCAGAGCTGGAAGGATTGGTAGT	gtcgtccc
FenA100-1	ctctcgggacgac	CGGTGAAAAGGACGGAATTCCTTTGCAACTAGT	gtcgtccc

The highlighted sequences represent candidate aptamers.

Table S4. Stock solutions of compounds for testing sensor cross-reactivity. All of the compounds were diluted in 1 mL of acetone and the working solution was diluted in 1x SB.

Compound	Concentration (mg/mL)	Concentration (mM)	Solvent
Fenitrothion	100	360.7113	Acetone
Malathion	100	302.7019	Acetone
Paraoxon	100	363.3761	Acetone
Parathion	100	343.2887	Acetone
Thioflavin T	1.0	3.1362	Methanol

Table S5. List of novel FAM sensors designed in this study.

No	Name of Sensor	Forward Primer	Sequence (5'->3')	Rev Primer	Number of copies
1	FenA1-FAM	FAM-CTCTCGGGACGAC	CACAGGGAGTAAGAGGCCGCCAGATTGTAA	GTCGTCCC	15
2	FenA2-FAM	FAM-CTCTCGGGACGAC	GGGCCGAGTAGTCTCCACGATTGATCGGAA	GTCGTCCC	14
3	FenA3-FAM	FAM-CTCTCGGGACGAC	CGCAGGTCAGGTTGAGAGGATCCCGATTGCT	GTCGTCCC	8
4	FenA4-FAM	FAM-CTCTCGGGACGAC	CGCAGGTTGTCTGAGCCGACAGGTTGCAT	GTCGTCCC	5
5	FenA5-FAM	FAM-CTCTCGGGACGAC	CGCAGGGCGTGAGAGCCGGAACGGTGCATC	GTCGTCCC	5

The red letter mean FAM molecules that modification at 5' end of each sensor

Table S6. List of novel ssDNA aptamers used for ThT sensor design.

No	Name of sensor	Forward Primer	Sequence (5' to 3')	Reverse Primer	Number of copies
1	FenA1-ThT	**ACGAC**	**CACAGGGAGTAAGAGGCCGCCAGATTGTAA**	**GTCGT**	15
2	FenA2-ThT	**ACGAC**	**GGGCCGAGTAGTCTCCACGATTGATCGGAA**	**GTCGT**	14
3	FenA3-ThT	**C**	**CGCAGGTCAGGTTGAGAGGATCCCGATTGCG**	**G**	8
4	FenA4-ThT	**ACGAC**	**CGCAGGTTGTCTGAGCCGACAGGTTGCAT**	**GTCGT**	5
5	FenA5-ThT	**ACGAC**	**CGCAGGGCGTGAGAGCCGGAACGGTGCATC**	**GTCGT**	5

Table S7. The list of all quenchers used in the FAM sensing assay.

Name	Sequence
Quencher 13 mer	5'- GTC GTC CCG AGA G /3'Dab/
Quencher 14 mer A	5'- AGT CGT CCC GAG AG /3'Dab/
Quencher 14 mer T	5'- TGT CGT CCC GAG AG /3'Dab/
Quencher 14 mer G	5'- GGT CGT CCC GAG AG /3'Dab/
Quencher 14 mer C	5'- CGT CGT CCC GAG AG /3'Dab/
